# In Vitro hRPTEC TERT1 Model for Uranium‐Induced Nephrotoxicity Pathway Study

**DOI:** 10.1155/jt/6692188

**Published:** 2026-06-17

**Authors:** Marie Frerejacques, Victoria Powell, Sebastien Giraud, Annabelle Manoury, Céline Bouvier-Capely, Thierry Hauet, Clara Steichen, Yann Guéguen

**Affiliations:** ^1^ LRSI, SESANE, PSE-SANTE, Nuclear Safety and Radiation Protection Authority (ASNR), Fontenay-aux-Roses, 92262, France; ^2^ Biochemistry Department, University of Poitiers, INSERM IRMETIST U1313 CHU of Poitiers, Poitiers, 86000, France, univ-poitiers.fr

**Keywords:** adverse outcome pathway, hRPTEC TERT1 cells, nephrotoxicity, uranium

## Abstract

As a heavy metal and alpha emitter, uranium U(VI) presents chemical and radiological toxicity risks. Its toxicity particularly targets the kidneys in the event of intoxication, but the detailed study mechanisms leading to uranium‐induced renal failure have not yet been studied based on the adverse outcome pathway (AOP) approach. Using a well‐specialized in vitro model of renal proximal tubule epithelial cells (hRPTEC TERT1), this research aims to contribute to the development of the AOP of kidney toxicity. After identifying the U(VI) concentrations that induce deleterious effects (apoptosis and necrosis/cytotoxicity), key events linked to oxidative stress, apoptosis, survival, inflammation, and kidney toxicity are studied at the gene and protein levels. Apoptosis (Caspase 3/7 activity) is induced starting from exposure to 300 μM and necrosis (LDH assay) from 500 μM with a rate of 60%. hRPTEC cells have an IC50 of 420 μM after 48 h U(VI) exposure. Uranium induces an early 2‐fold rise in ROS production, and an antioxidant response was observed accordingly. Cell survival signaling appeared to be enhanced at 100 μM, while higher concentrations (> 300 μM) induced a marked inflammatory response, with increased levels of TNFα (6,5‐fold), IL‐6, and IL‐18, leading to a 16% increase in caspase 3/7 at 300 μM and LDH at 500 μM. A slight increase of 22% in the KIM‐1 protein level is observed at 500 μM, and limited changes occurred for other nephrotoxicity biomarkers. In conclusion, this exploratory work generated a multiplex panel detailing the key events in the AOP of uranium‐induced renal failure (Graphical Abstract). This panel features a human phenotype of renal proximal tubule epithelial cells, the preferential target of uranium in the kidney, while also considering the dose‐dependent effects.

**Highlights**
• IC50 U (VI) cytotoxicity is defined at 420 μM on hRPTEC TERT1
• U (VI) causes an increase of ROS and altered antioxidative enzymes expression
• Cell survival appeared to be enhanced at 100 μM U (VI)
• Inflammatory response is triggered at 500 μM U (VI) with a rise in TNFα, IL‐6, and IL‐18
• KIM‐1 is increased at 500 μM U (VI), other nephrotoxicity markers slightly changed.

## 1. Introduction

Uranium is a heavy metal and a radioactive element that emits alpha particles, making it both chemically and radiologically toxic. It is naturally found in the environment, in soils [1, 2], and water [3, 4] and is also used in human activities such as the nuclear fuel cycle [5, 6] and the manufacturing and use of weapons [7], which can lead to occupational or environmental exposure. Epidemiological studies have shown that environmental or professional exposure to uranium can lead to health problems [8, 9] and particularly kidney toxicity and failure [4, 10–12]. Regardless of the exposure route and dose, uranium is known to cause renal tubular damage and impair kidney function [13]. Indeed, uranium specifically damages the proximal tubules due to its reabsorption by epithelial cells during filtration by the kidney [14–16].

The initiation of molecular key events by uranium exposure starts with mitochondrial electron transport chain (ETC) inhibition, resulting in mitochondrial disruption as shown in rat kidney cells [17, 18]. The generation of free radicals (ROS) results in reduced levels of glutathione, as well as diminished activity of catalase (CAT) and superoxide dismutase (SOD) enzymes in renal cells [19–21], ultimately causing oxidative stress.

The activation of Nrf2 probably contributes to endoplasmic reticulum stress, which, in turn, contributes to caspase activation and apoptosis. Mitochondrial disruption further influences survival and apoptosis mechanisms by modulating signaling pathways such as PI‐3K, MAPK, Bax/Bcl‐2, and Fas/Fas L and regulating anti‐/proapoptotic factors such as Bcl‐2‐ and Bcl‐2‐associated proteins. The NF‐kB inhibition is also described after U‐exposure, resulting in increased production of TNFα demonstrated in a macrophage cell line [22].

Uranium exposure then induces apoptosis through caspase activation, while necrosis is associated with the heightened release of enzymes such as lactate dehydrogenase (LDH), demonstrated on various human kidney cell models [23–25]. On animal models, kidney toxicity due to uranium exposure manifests as alterations in renal function indicators, including serum creatinine, blood urea nitrogen, alkaline phosphatase, B2‐microglobulin, LDH, and N‐acetylglutamate [26, 27]. These changes indicate tubular damage and dysfunction in kidney filtration function, ultimately leading to kidney failure.

These mechanisms of uranium‐induced kidney toxicity have been recently compiled in the form of an adverse outcome pathway (AOP), consolidating the existing body of knowledge from in vitro, in vivo, and epidemiological data and highlighting the need to fill the gaps in understanding of the U‐toxicity mechanisms [28]. In the field of radiation protection, the study of uranium’s renal toxicity is strongly encouraged through the AOP concept, being promoted as a priority [29]. The development of an AOP serves to highlight knowledge gaps and prompts prioritization of research efforts aimed at assessing the health risks associated with exposure to physical or chemical stressors [30, 31]. Its development has been strongly encouraged by the Organization for Economic Co‐operation and Development (OECD) in the field of chemical risk assessment [32] and is applied to other areas requiring risk assessment as in the field of radiation protection [33, 34]. However, there is a need to supplement and refine the existing knowledge, by delving deeper into the underlying mechanisms of toxicity with the use of an accurate human model of epithelial cells from the proximal tubules.

The human hRPTEC TERT1 model exhibits many characteristics of its in vivo counterparts, such as specific transporter expression, tight junction formation, polarization with apical and basolateral membrane, γ‐glutamyl transferase activity, and high genetic stability [35–38]. hRPTEC TERT1 has already proved useful in assessing the toxicity of heavy metals such as cadmium [39], as well as other nephrotoxic agents such as cisplatin, cyclosporin, aristolochic acid I, and gentamicin [40–42]. These studies have notably shown that nephrotoxicity biomarkers such as KIM‐1, B2 microglobulin, clusterin, and cystatin C, typically studied in vivo, were relevant in this renal cellular model.

Therefore, for studying uranium‐induced nephrotoxicity, the hRPTEC TERT1 model appears to be an ideal candidate offering a valuable alternative to currently used renal cell models. Its properties provide an opportunity to improve the understanding of toxicity mechanisms and contribute to the development of the AOP for renal failure. To investigate uranium toxicity, the research will focus on dose‐dependent uranium cytotoxicity to define subtoxic and toxic doses necessary for studying cellular events. This study aims to investigate mechanisms related to previously identified key events: oxidative stress, apoptotic and cell survival mechanisms, inflammation, and kidney injury at mRNA, protein, and enzyme activity levels.

## 2. Materials and Methods

### 2.1. Cell Culture

The hRPTEC TERT1 cells (ATCC, CRL‐4031) were maintained in a DMEM/F12 medium (Cytivia), supplemented with 10 ng/mL of hEGF, 0.1 mg/mL of G418, 1% ITSG, 3.5 μg/mL of ascorbic acid, and 25 ng/mL of prostaglandin E1 (ATCC). Cells were seeded in 6‐well plates with a density of 500,000 cells per well and in 96‐well plates with a density of 40,000 cells per well for uranium exposure. Cultures were maintained under controlled conditions in a humidified atmosphere with 95% air and 5% CO_2_ at a temperature of 37°C.

### 2.2. Uranium Exposure

A 10 mM U‐bicarbonate stock solution (U solutions) was prepared by dissolution of uranyl nitrate (UO_2_(NO_3_)_2_, 6H_2_O; ^238^U: 99.74%, ^235^U: 0.26%, ^234^U: 0.001%, Merck‐Prolabo) in 100 mM NaHCO_3_. The stock solution was stirred for 5 min to ensure the total dissolution of the crystals and filtered through 0.22‐μm filter units. For each experiment, cells were exposed to uranyl (U(VI)) solutions (0–1000 μM) diluted in the DMEM/F12 medium for 48 h. The level of uranium in solutions was attested by ICP‐MS (iCAP RQ, Thermo Fisher Scientific). After appropriate dilution in 2% nitric acid, uranium was quantified with bismuth as an internal standard and a uranium external calibration curve. The detection limit of uranium measurement by ICP‐MS is 0.5 ng/L for ^238^U and 0.01 ng/L for ^235^U.

### 2.3. Cell Viability Tests

#### 2.3.1. LDH Assay

Cell viability was determined by measuring LDH in supernatant after 2–48 h of uranium exposure, using LDH‐Glo Cytotoxicity assay (Promega) according to the manufacturer’s instructions. Measurements were made by spectrophotometer (TECAN SPARK, TECAN). The percentage of viability was determined as extracellular LDH of treated cells versus positive cytotoxicity control cells obtained by an exposure to Triton 1% (Sigma Aldrich) for 15 min.

#### 2.3.2. Caspase 3/7 Assay

Apoptosis was determined by measuring intracellular caspase 3/7 after 24 or 48 h of uranium exposure, using Caspase‐Glo 3/7 assay (Promega) according to the manufacturer’s instructions. Measurements were made by spectrophotometer (TECAN SPARK, TECAN). The percentage of apoptosis was determined as intracellular Caspase 3/7 of treated cells versus positive apoptosis control cells obtained by exposure to Staurosporine 10 μM solution (Sigma Aldrich) for 24 h.

### 2.4. ROS Assessment

The reactive oxygen species (ROS) were assessed by the ROS‐Glo H_2_O_2_ Assay (Promega) according to the manufacturer’s protocol. ROS luminescence was measured at 6, 18, 24, and 48 h after U(VI) exposure for a kinetics study.

### 2.5. Real‐Time RT‐PCR

Total RNA was extracted from a cell pellet of one million cells using the RNeasy total RNA isolation kit (Qiagen) and reverse‐transcribed into cDNA using the High‐capacity cDNA reverse transcription kit (Thermo Fisher Scientific) according to the manufacturer’s instructions. Real‐time PCR was used to analyze the mRNA levels of NRF2, HO1, NQO1, CAT, OPN, SOD1, CASP8, BAX, BCL2, and KIM1 with SYBER Green technology (Thermo Fisher Scientific). Sequences for the forward and reverse primers used are listed in Table 1, and SOD2, LCN2, GCLC, CYP2E1, PSMA6, PSMB7, HSPA5, CHOP, ITRP2, ATP1A1, CBS, BSE, TNF, iNOS, IL‐6, COX2, PECAM1, GAL3, B2M, and CLU with Taqman technology (Thermo Fisher Scientific) using GAPDH as the housekeeping gene.

**TABLE 1:** Primer sequences.

Gene	Primers	5′–3′ sequence	Genebank reference
BAX	Forward	GCCGCCGTGGACACA	NM004324
	Reverse	TTGCCGTCAGAAAACATGTCA	

BCL2	Forward	AGAAGAGACTCTTTGCATATGACTCACA	NM000633
	Reverse	TCCTATGATTTAAGGGCATTTTTCC	

CASP8	Forward	CCTGGGTGCGTCCACTTT	NM001080
	Reverse	TCCCAAGGTTCAAGTGACCAA	

CAT	Forward	CGCCTGGACCCAATTATCT	NM_001752.3
	Reverse	GCCGTCACGCTGGTACTTG	

GAPDH	Forward	TCAACGGATTTGGTCGTATTG	NM_001289745.1
	Reverse	TCTCGCTCCTGGAAGATGG	

HO1	Forward	TACCGCTCCCGCATGAA	NM_002133.2
	Reverse	CGCAGTCTTGGCCTCTTCTATC	

KIM1	Forward	CTTCACCTCAGCCAGCACAAAC	NM_001173393.3
	Reverse	GCCATCTGAAGACTCTGTC	

NQO1	Forward	GGTTTGAGCGAGTGTTCATAGG	NM_000903.3
	Reverse	GCAGAGAGTACATGGAGCCAC	

NRF2	Forward	CTCCTACACCAACGCCTTTCC	NM_006164.4
	Reverse	GGAGTTCGGACGCTTTGAAAC	

OPN	Forward	TCACCAGTCTGATGAGTCTCAC	AF052124
	Reverse	CAGGTCTGCGAAACTTCTTAGAT	

SOD1	Forward	GGGCAATGTGACTGCTGACA	NM_000454
	Reverse	TGCGGCCAATGATGCA	

### 2.6. Protein Assays

#### 2.6.1. Proteome Profiler Arrays

The supernatant was analyzed using the Proteome Profiler Human Cytokine Array and Kidney Biomarker (R&D Systems). Cell homogenates were analyzed using the Proteome Profiler Apoptosis Array and Cell Stress Array (R&D Systems). Protein levels were visualized with enhanced chemiluminescence on an imaging system (Amersham ImageQuant 800, CYTIVIA). Intensities of signals were quantified using Cytivia software.

#### 2.6.2. Multiplex Immunoassay

The inflammation markers (IL‐1β, IL‐1ra, IL‐6, IL‐10, IL‐18, TNFα) and the nephrotoxicity markers (Calbindin, GSTa, TIMP1, KIM‐1, IP10, Renin, Collagen IV, TFF3) were measured in the supernatant with a 1:1 dilution using Milliplex plates according to the manufacturer’s instructions (Merck Millipore).

### 2.7. Statistical Analysis

Normality of the data was tested. In the absence of normality, Tukey or Holm–Sidak testing was used to conduct a one‐way analysis of variance (ANOVA) considering the different concentrations of U(VI) and a two‐way ANOVA considering the time of exposure and the concentrations of U(VI) (SigmaPlot 15.0 Software). The results were considered statistically at *p* < 0.05. The *n* value was specified in the legends of each figure.

## 3. Results

### 3.1. Dose‐Dependent Cell Apoptosis and Necrosis of hRPTEC TERT1 Exposed to U (VI)

**FIGURE 1:**
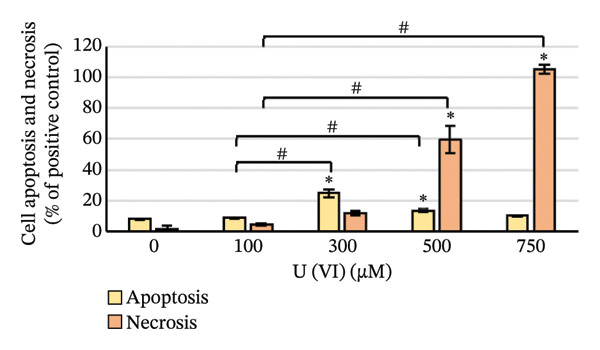
Effect of uranium on apoptosis and necrosis for hRPTEC TERT1 cells. Cells were treated with different concentrations of uranium for 48 h. Next, cell apoptosis was measured by caspase 3/7 luminescence assay and cell necrosis was measured by LDH luminescence assay. Data were expressed as a percentage of the positive control, treatment with staurosporine 10 μM over 24 h for apoptosis induction, and treatment with triton 1% for 15 min for necrosis induction. Data were expressed as mean ± SEM where *n* = 8 per condition. Asterisk represents a significant difference between U (VI)‐treated and untreated cells (two‐way ANOVA ^∗^ *p* < 0.05; ^∗∗^ *p* < 0.01; ^∗∗∗^ *p* < 0.001); sharp sign represents a significant difference between U (VI)‐treated concentrations (two‐way ANOVA ^#^ *p* < 0.05; ^##^ *p* < 0.01; ^###^ *p* < 0.001).

To evaluate the adverse effect of U(VI) exposure over 48 h in hRPTEC TERT1 cells, the dose‐dependent cytotoxicity was measured according to apoptosis and necrosis hallmarks (e.g. Caspase 3/7 and LDH) (Figure 1). A significant increase of caspase 3/7 apoptotic enzyme starts from 300 μM of exposure to U(VI). In fact, the apoptosis rate is 24.7 ± 2.4% (*p* < 0.05) for 300 μM U (VI) and represents a 16% significant increase compared to the basal cell apoptosis rate on control. Exposure to 500 μM of U(VI) induces a lower apoptosis rate of 13.6% ± 1.2% (*p* < 0.05), showing a significant 5% increase compared to the control. Exposure to the highest dose of 750 μM U(VI) does not induce significant change in apoptosis evaluated by caspase 3/7 activity, probably due to necrotic effect at this high concentration. Caspase 3/7 activity showed a minimal increase following 24 h of U(VI) exposure at 500 μM (6% of positive control) and 1000 μM (8% of positive control) (Supporting Figure S1).

LDH release is correlated to loss of membrane integrity and therefore necrosis, either primary necrosis or secondary necrosis (following apoptosis for instance). A significant increase of LDH release was observed only after exposure to 500 μM of U(VI), with a total necrosis rate of 59.6 ± 8.6% (*p* < 0.05) for the cell population of hRPTEC TERT1. After exposure to 750 μM of U(VI), the entire cell population is necrotic. The IC50 of uranium U(VI) for hRPTEC TERT1 cells is estimated to be 420 μM after 48 h of exposure. No significant cytotoxic effect was observed before 48 h of exposure below 1000 μM of uranium, as indicated by the absence of elevated LDH levels after 24 h of U(VI) exposure at low concentrations (Supporting Figure S1).

It is suggested that an apoptotic mechanism is initiated at a subtoxic dose of 300 μM, while the necrotic mechanism becomes predominant beyond the IC50 of 420 μM. As a result, studies were conducted at 48 h of exposure, maintaining concentrations without adverse effects (100 μM), with an apoptotic effect (300 μM), and with a necrotic effect (500 μM) to explore pathways identified in the preliminary AOP of kidney failure.

### 3.2. Modification of the Apoptosis or Survival Pathway

**FIGURE 2:**
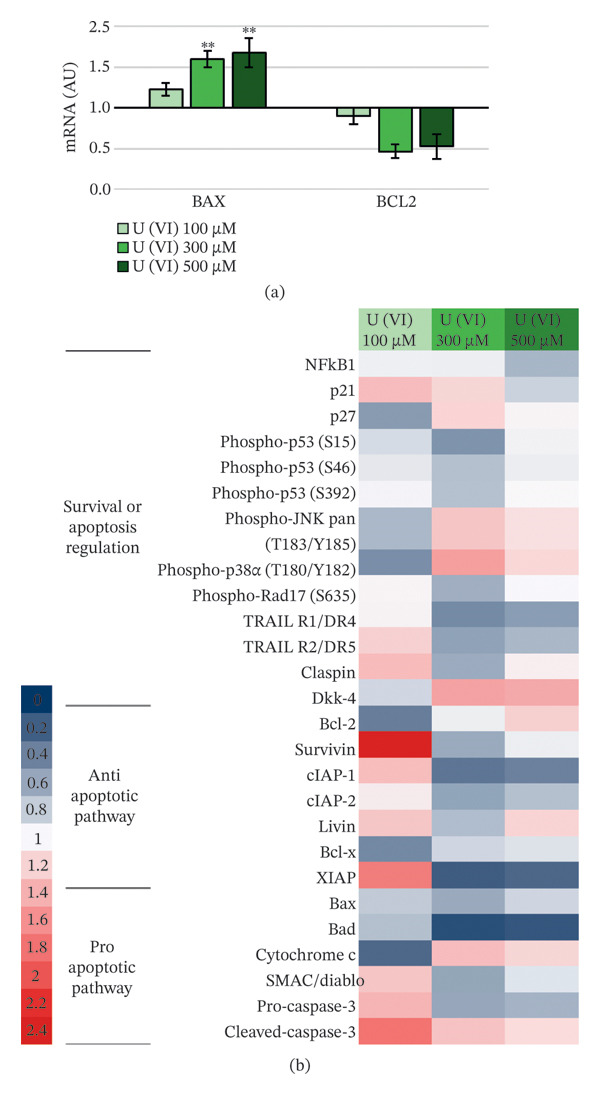
Effect of uranium on apoptosis or survival pathway for hRPTEC TERT1 cells. Cells were treated with different concentrations of uranium for 48 h. (a) Screening of gene expression implicated in apoptosis or survival pathway. Data were expressed as mean ± SEM (*n* = 8 for 0, 100 and 300 μM; *n* = 5 for 500 μM). (b) Proteome profiler array on apoptosis or survival pathway. Results are expressed as a chemiluminescence signal ratio (treated to untreated cells) in the form of a heat map (*n* = 1 per condition). Asterisk represents a significant difference between U (VI) treated and untreated cells (two‐way ANOVA ^∗^ *p* < 0.05; ^∗∗^ *p* < 0.01; ^∗∗∗^ *p* < 0.001); sharp sign represents a significant difference between U (VI)‐treated concentrations (two‐way ANOVA ^#^ *p* < 0.05; ^##^ *p* < 0.01; ^###^ *p* < 0.001).

**FIGURE 3:**
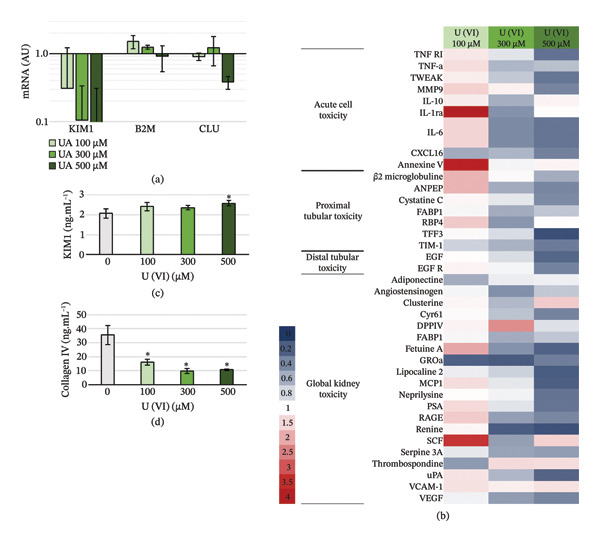
Effect of uranium on nephrotoxicity biomarkers for hRPTEC TERT1 cells. Cells were treated with different concentrations of uranium for 48 h. (a) Gene expression of nephrotoxicity biomarkers. Data were expressed as mean ± SEM (*n* = 3 per condition). (b) Proteome profiler array on nephrotoxicity. Results are expressed as a ratio of untreated to treated cells at different concentrations in the form of a heat map (*n* = 1 per condition). (c) KIM1 protein assay by Milliplex (LOQ 0.05 ng.mL^−1^). Data were expressed as mean ± SD (*n* = 3 per condition). (d) Collagen IV protein assay by Milliplex (LOQ 0.5 ng.mL^−1^). Data were expressed as mean ± SD (*n* = 3 per condition). Asterisk represents a significant difference between U (VI)‐treated and untreated cells (two‐way ANOVA ^∗^ *p* < 0.05; ^∗∗^ *p* < 0.01; ^∗∗∗^ *p* < 0.001).

To further explore the mechanisms of apoptosis leading to the elevation of caspase 3/7 after 300 μM U(VI) exposure (Figure 1), the expression of two proteins involved in the regulation of apoptosis, BAX and BCL2, was investigated (Figure 2a). Only the BAX expression is modified with an increased mRNA level from 300 to 500 μM, compared to the 100 μM condition (fold change of 1.6 ± 0.1 (*p* < 0.01) and 1.7 ± 0.2 (*p* < 0.01), respectively, compared to the 100 μM condition). Moreover, the measurement of annexin V level, a classical hallmark of apoptosis, was performed via a membrane array rather than by Annexin V/PI flow cytometry (Figure 3). This constitutes a potential limitation of the current study design.

To obtain an overall view of the impact of U(VI) exposure on the pro‐ or antiapoptotic response, screening with a proteome profiler was undertaken, and classified as “survival or apoptosis regulation,” “anti‐apoptotic pathway,” and “proapoptotic pathway” (Figure 2b). The exploratory data obtained showed that the level of “survival and apoptosis regulation” proteins in this classification is repressed or unmodified at 78%, 57%, and 71%, respectively, at 100, 300, and 500 μM. The following protein levels were detected by this protein array only: p27, implicated in the maintenance of genetic stability and the regulation of apoptosis, is strongly repressed only at 100 μM, and p53 (S15), associated with mediating growth arrest and sensitizing cells to apoptosis, is repressed at 300 μM; p38, regulating survival, is strongly repressed at 100 μM and then overexpressed at 300 μM. Regarding the “anti‐apoptotic pathway,” BCL2 is repressed at 100 μM and minimally modified at other concentrations; at 100 μM, Survivin, and XIAP, inhibitors of apoptosis, are overexpressed. It is important to note that the “antiapoptotic pathway” is overexpressed in 57% of the targets only at 100 μM, and then at 300 and 500 μM, it is repressed at 86% and 57%, respectively. As for the “proapoptotic pathway,” it is important to note that although the gene expression of BAX is increased (Figure 2a), in protein expression, BAX is minimally modified, even underexpressed. The proapoptotic pathway is overexpressed for 50% of its targets at 100 μM, and it is minimally modified or strongly repressed, especially for BAD, at 300 and 500 μM (Figure 2b).

### 3.3. Uranium Causes an Increase of ROS and Altered Antioxidative Enzyme Expression

**FIGURE 4:**
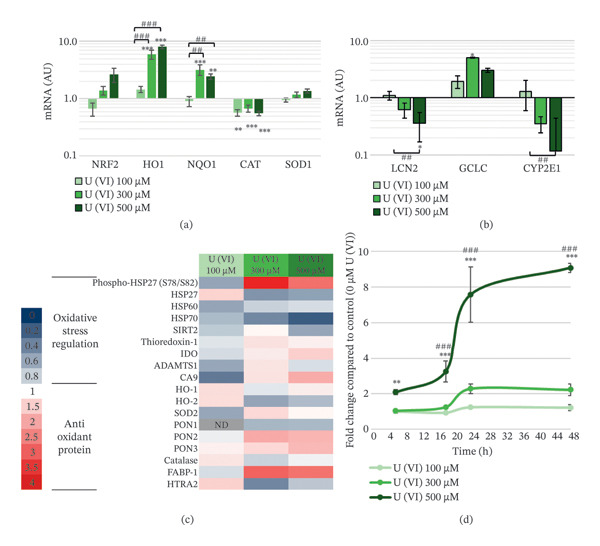
Effect of uranium on oxidative stress for hRPTEC TERT1 cells. Cells were treated with different concentrations of uranium (U (VI)) for 48 h. (a) Gene expression of antioxidant enzymes. Results are expressed as a ratio to the expression of the housekeeping gene GAPDH. Data were expressed as mean ± SEM (*n* = 8 for 0, 100, and 300 μM; *n* = 5 for 500 μM). (b) Gene expression of targets implicated in the oxidative stress pathway. Results are expressed as a ratio to the expression of the housekeeping gene GAPDH. Data were expressed as mean ± SEM (*n* = 3 per condition). (c) Proteome profiler array on oxidative stress pathway. Results are expressed as chemiluminescence signal ratio (U (VI) treated to untreated cells) in the form of a heat map (*n* = 1 per condition). (d) Kinetics of reactive oxygen species production after exposure to U (VI) for 48 h (*n* = 3 per condition). Asterisk represents a significant difference between U (VI)‐treated and untreated cells (two‐way ANOVA ^∗^ *p* < 0.05; ^∗∗^ *p* < 0.01; ^∗∗∗^ *p* < 0.001); sharp sign represents a significant difference between U(VI)‐treated concentrations (two‐way ANOVA ^#^ *p* < 0.05; ^##^ *p* < 0.01; ^###^ *p* < 0.001).

Oxidative stress generated by the increase in ROS due to U‐exposure is one of the earliest events observed. ROS quantification was performed as a function of time (6, 18, 24, and 48 h) and concentrations (0, 100, 300, and 500 μM) (Figure 4d). A significant increase in ROS at 500 μM of uranium after as early as 6 h of exposure was observed. Compared to the control unexposed cells, exposure to 500 μM of U(VI) increased the ROS level (fold change of 2.0 ± 0.2 (*p* < 0.004) at 6 h, 3.2 ± 0.6 (*p* < 0.001) at 16 h, 7.6 ± 1.6 (*p* < 0.001) at 24 h, and 9.1 ± 0.3 (*p* < 0.001) at 48 h). It is important to note that although there was no significant increase compared to the control at 100 and 300 μM, the ROS levels quantified at these doses were significantly different from 500 μM (*p* < 0.001). Additionally, ROS levels at 500 μM were statistically different from those observed at 300 μM and 100 μM (*p* < 0.001) across all exposure durations (18, 24, and 48 h) (Figure 4d).

The expression of genes coding for antioxidant enzymes, identified as playing a key role in oxidative response, was studied after 48 h of U(VI) exposure with 100, 300, or 500 μM concentrations (Figure 4a). It can be observed that the expression of heme oxygenase‐1 (HO1) increased significantly from 300 μM of U(VI) exposure, with a fold change of 4.4 ± −0.4 (*p* < 0.001), and further increased to a fold change of 6.2 ± 0.5 (*p* < 0.001) at 500 μM. Similarly, the expression of NAD(P)H quinone dehydrogenase 1 (NQO1) showed an increase from 300 μM, with a fold change of 2.4 ± 0.2 (*p* < 0.001), and at 500 μM, with a fold change of 1.8 ± 0.2 (*p* < 0.001). Notably, a dose–response effect is observed between the two highest doses and the dose of 100 μM (*p* < 0.001) for the genes HO1 and NQO1, whereas the response is similarly increased for the highest dose. Nevertheless, the NRF‐2 gene expression was minimally induced, yet the effect was not statistically significant. In contrast, the expression of CAT was repressed for all doses, with respective fold changes of 0.6 ± 0.06 (*p* < 0.01), 0.5 ± 0.03 (*p* < 0.001), and 0.4 ± 0.06 (*p* < 0.001). No significant change of the gene expression of superoxide dismutase 1 (SOD1) was observed.

Other targets playing a role in pro‐ and antioxidant balance and involved in the cell response to uranium have been investigated in gene expression (Figure 4b). The gene expression of lipocalin‐2 (LCN2) was significantly repressed starting only at 500 μM, with a fold change of 0.4 ± 0.2 (*p* < 0.05), a 2.5‐fold repression. For glutamate‐cysteine ligase catalytic (GCLC) subunit, a significant increase was only observed after exposure to 300 μM, with a fold change of 4.9 ± 0.1 (*p* < 0.05). Regarding the gene expression of cytochrome P450 2E1 (CYP2E1), the CYP2E1 mRNA level decrease was not significant for the 300 μM condition compared to the control, but a decreased mRNA level was observed with a fold change of 10 ± 0.3 (*p* < 0.01) for the 500 μM concentration compared to the lowest tested concentration of 100 μM.

To obtain an overall view of the impact of U(VI) exposure, a screening of the expression of proteins for oxidative stress was conducted (Figure 4c). The exploratory data obtained by protein array only suggested that proteins involved in “oxidative stress regulation” are repressed at 88% after exposure of hRPTEC cells to 100 μM of exposure U(VI), while “antioxidant proteins” were overexpressed at 55%. Exposure to 300 μM impacted the “oxidative stress regulation” resulting in an overexpression of 44% and impacted also the “antioxidant proteins” with an overexpression of 44%. For exposure to 500 μM, the “regulatory pathway” and the “antioxidant proteins” remained globally impacted, with 33% of overexpression. Overall, it is interesting to note that proteins involved in the regulation of oxidative stress and antioxidant proteins appeared to be overexpressed from 300 μM of uranium after 48 h of exposure, whereas lower concentrations induced a low diminution of their protein level.

### 3.4. U (VI) Exposure Induces the Inflammatory Response

**FIGURE 5:**
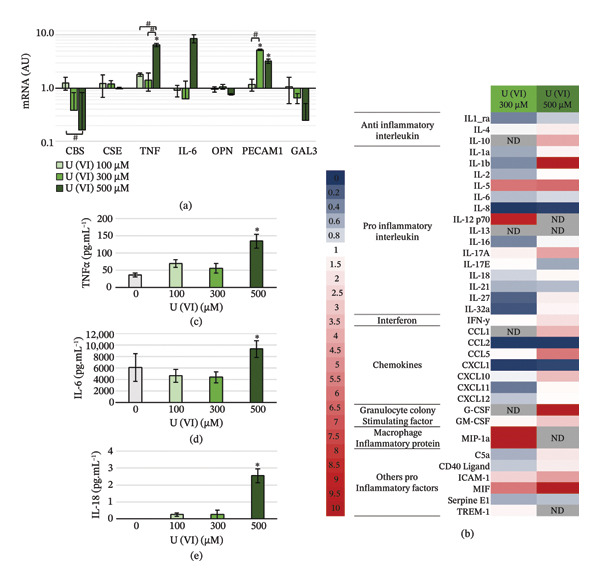
Effect of uranium on inflammation for hRPTEC TERT1 cells. Cells were treated with different concentrations of uranium for 48 h. (a) Screening of gene expression implicated in the inflammation pathway. Data were expressed as mean ± SEM (*n* = 3 per condition). (b) Proteome profiler array on inflammation pathway. Results are expressed as a ratio of untreated to treated cells at different concentrations in the form of a heat map (*n* = 1 per condition). (c) TNFα protein assay by Milliplex. Data were expressed as mean ± SD (*n* = 3 per condition). (d) IL‐6 protein assay by Milliplex. Data were expressed as mean ± SD (*n* = 3 per condition). (e) IL‐18 protein assay by Milliplex. Data were expressed as mean ± SD (*n* = 3 per condition). Asterisk represents a significant difference between U (VI)‐treated and untreated cells (two‐way ANOVA ^∗^ *p* < 0.05; ^∗∗^ *p* < 0.01; ^∗∗∗^ *p* < 0.001); sharp sign represents a significant difference between U (VI)‐treated concentrations (two‐way ANOVA ^#^ *p* < 0.05; ^##^ *p* < 0.01; ^###^ *p* < 0.001).

The effect of U(VI) on cell inflammation was further investigated at the gene and protein expression level as well as the protein secretion level. Regarding the mRNA levels of some target genes (Figure 5a), cystathionine synthase (CSE), OPN, and GAL3 show no variation in their gene expression. CBS was reduced 5‐fold (*p* < 0.05) only at a concentration of 500 μM compared to the lowest concentration of 100 μM. TNFα increased 6.5‐fold (*p* < 0.05) at 500 μM compared to the control. For IL‐6, a nonsignificant increase was observed after exposure to 500 μM with a fold change of 8.6 ± 1.5. PECAM 1 was increased from 300 μM with a fold change of 5.2 ± 0.1 (*p* < 0.05) and at 500 μM with a fold change of 3.2 ± 0.3 (*p* < 0.05).

The screening of the inflammatory process was conducted on the cell supernatant with a proteome profiler array to obtain anoverall response of the pathway with semi‐quantitative data (Figure 5b). The data obtained by the protein array only possibly showed that this pathway was strongly altered at the two concentrations tested: 53% of the protein targets are repressed at 300 μM, and only 14% are overexpressed. An increase in the number of overexpressed targets was observed at 500 μM, reaching 35%. Moreover, this is the pathway with the strongest effects observed. All these findings indicate that this pathway appears to be preferentially modified after 48 h of exposure to 500 μM of uranium.

In this sense, important cytokines identified in the cellular response to uranium have been precisely quantified by ELISA: TNFα showed a threefold increase (*p* < 0.05) at 500 μM (Figure 5c); IL‐6 was also increased twofold (*p* < 0.05) at the dose of 500 μM (Figure 5d); and IL‐18 was also increased twofold (*p* < 0.05) at 500 μM (Figure 5e). The concentrations of IL1‐β, IL‐1ra, and IL‐10 in the cell supernatant were below the limit of quantification by Milliplex assay.

### 3.5. Low Changes of Nephrotoxicity Biomarkers

The gene expression of KIM‐1, B2M, and CLU was not significantly modified (Figure 3a). The exploratory data obtained only by the protein profiler array of nephrotoxicity biomarkers (Figure 3b) showed the targets of cellular and renal damage that are overexpressed by 42% at 100 μM, with a strong overexpression for IL‐1ra, Annexin V, B2M, ANPEP, Fetuin A, and SCF. From 300 μM of U(VI), the targets of this damage were more extensively decreased, between 66% and 71%, with only DPPIV, a marker of the overall renal damage, strongly overexpressed at 300 μM.

Important kidney biomarkers have been precisely identified by ELISA in the cell supernatant: KIM‐1 was only slightly increased by 22% (*p* < 0.05) at 500 μM (Figure 3c). Collagen IV decreased by nearly 3 times (*p* < 0.05) from 100 μM to 500 μM (Figure 3d). The concentrations of TFF3, calbindin, GSTa, and IP‐10 in the cell supernatant were below the limit of quantification by the Milliplex assay.

## 4. Discussion

To improve the understanding of U‐nephrotoxicity mechanisms through the development of the AOP for renal failure that has been previously proposed (https://aopwiki.org/aops/447), the hRPTEC TERT1 was used as a model of U‐targeted cells. These cells maintained many hallmarks of in vivo RPTEC compared to other renal epithelial cell lines. The U(VI) concentrations used in our study exceed environmental exposure levels. However, the range includes low to moderate doses and accounts for the uncertainty factor related to the highly heterogeneous distribution of uranium within the kidneys of exposed in vivo models. In vivo studies show that the site‐specific accumulation of uranium can reach 1000–3000 μg/g (i.e., 2–6 mM). These concentrations are 10‐ to 100‐fold higher in the cortical region (which contains the proximal convoluted tubules, or PCTs) than the mean renal tissue level [28, 43].

The initial step of our work was to define a concentration of U(VI) that would cause a measurable adverse effect or adverse outcome (AO). The IC50 on hRPTEC was established at 420 μM U(VI) after 48 h of exposure, and their sensitivity appeared similar than that of other human or animal renal cell cultures used in the assessment of U cytotoxicity: between 600 μM [23] and 820 μM [24] after 24 h for HK2; 630 μM after 48 h for HK2 [24]; 500 μM after 24 h for HEK293 [25]; 800 μM after 24 h for LCCPK1 [44]; and 500 μM after 24 h for NRK‐52E [45]. Identification of the IC50 allowed us to define the concentrations to study the mechanisms of adverse effects induced by U(VI) with moderate or low cell death (< 500 μM). The biological sequence of events observed at concentrations below 300 μM diverges from that observed at higher, cytotoxic concentrations (i.e., apoptosis and cytotoxicity/cell membrane damages). Consistent with findings in other cellular models, the cellular response to noncytotoxic concentrations (< 100 μM) is probably indicative of cellular stress adaptive response mechanisms [46].

Exposure to uranium U(VI) at a concentration of 100 μM does not induce significant oxidative stress, as evidenced by the absence of ROS induction. Consequently, no antioxidant response was observed, but antiapoptotic response could be induced (or begins to be induced) as suggested by our data from the proteome profile array showing an increased protein level in apoptosis inhibitors such as XIAP and Survivin, with no elevation of caspase 3/7 activity, the enzymes involved in triggering apoptosis. No proapoptotic response with caspase 3/7 elevation had been observed at this low concentration in other in vitro models previously used, like the rat renal epithelial cell line NRK52 [47, 48]. Accordingly, an anti‐inflammatory response is probably induced, as shown by the elevated IL1‐ra protein level, a natural inhibitor of IL‐1β, and with no change of proinflammatory cytokines such as TNFα, IL‐6, and IL‐18 measured by ELISA. Proinflammatory response with elevation of TNFα was noted on the primary and cell line of rat alveolar macrophage after 24 h exposure to U(VI), respectively, at 100 μM [49] and 50 μM U(VI) [22]. In this context, a specific cellular type was involved in the inflammatory response, distinct from tubular proximal epithelial cells, which, although implicated in the phenomenon of inflammation, do not play a major role. Consequently, a delayed response to a stronger exposure can be expected. As for markers of global renal damage analyzed only from the proteome profile array, an elevation of SCF (stem cell growth factor) is accompanied by an increase in metalloproteinase 9 (MMP9). Metallothioneins (MTs) have a metal‐binding function that involves them in the detoxification of heavy metals and essential metal homeostasis. By interacting with zinc ions, they may protect cells from oxidative stress, or apoptosis caused by U exposure, as reported in HK‐2 [19]. SCF and MMP9 are known to act together to prevent apoptosis during acute kidney injury in mouse models [50]. The absence of cell death induced at this relatively low concentration of uranium could be correlated to predominant and effective cell protection mechanisms as previously shown with uranium exposure and other metals [46, 51, 52]. This suggests that hRPTEC cells have developed mechanisms to counteract the potentially harmful effects of uranium at this concentration.

Conversely, adverse effects such as apoptosis (300 μM) and cytotoxicity (LDH release) (500 μM) are induced when hRPTEC cells are exposed at higher concentrations (> 300 μM) of U. The mechanisms of cell death due to U‐exposure are correlated to oxidative stress and mitochondrial disruption that could lead to an intense increase in ROS, starting 6 h after U‐exposure, causing alterations in antioxidant enzymes as an increase in HO1 and NQO1 (Figure 3) [17, 18, 21]. Nevertheless, while NRF2 protein activation/nuclear translocation was not directly assayed, indirect analysis of the antioxidant response (via target genes) suggests it may be induced. A decrease in CAT activity was also observed in vitro [19, 20] and in vivo after exposure to U(VI). These antioxidant enzymes contribute to reducing the local oxidative stress. FABP1 is highly upregulated in hRPTEC and acts as an antioxidant protecting cells from oxidative stress and reducing cytotoxicity because it contains amino acids such as methionine and cysteine, which are known to be effective antioxidant agents [53]. Additionally, the strong repression of LCN2 could promote oxidative stress by inducing iron accumulation [54]. Expression of LCN2 suppressed hydrogen peroxide–induced apoptosis and prolonged cell survival, suggesting an antioxidative role for LCN2 [55]. Furthermore, Krejsa et al. [56] reported an increase in the expression of glutamate‐cysteine ligase (GCLC), following oxidative stress. GCLC plays a crucial role in the synthesis of glutathione, an important cellular defense mechanism against oxidative stress, previously described as downregulated in the kidneys of rats exposed to U at 5 mg/kg [57]. The decreased CYP2E1 protein level, measured by protein array and mRNA expression in our model, a cytochrome P450 enzyme involved in detoxifying xenobiotics, has been previously reported in the kidneys of U‐exposed rats [58] and is involved in the generation of ROS by redox recirculation due to its reductive activation of U(VI) [17]. Concerning regulation of oxidative stress, data obtained from the proteome profile array only suggested a repression of HSP60 and HSP70 and overexpression of phospho‐HSP27 that could be correlated to promoting cell survival and inhibiting apoptosis. The upregulation of HSP27 is a biomarker of kidney injury and considered a sign of rescue to prevent cell death [59]. All these key events indicate that a strong antioxidative response takes place at these cytotoxic concentrations but is probably not sufficient to counteract cell death.

The proapoptotic response occurs starting at 300 μM U(VI) due to caspase 3/7 elevation, as reported in in vitro and in vivo studies on U cytotoxicity, in kidney tissue and kidney cells, as well as in other cell types [17, 18, 25, 60–62]. This apoptotic activity is attributed to lysosomal or mitochondrial alterations, which increases ROS production in rat renal or HepG2 cells [17, 18] as seen on hRPTEC TERT1 (Figure 4). At 500 μM, a strong protein repression of XIAP and cIAP‐1 evaluated by protein array only, inhibitors of apoptosis, could lead to a proapoptotic response. Also noteworthy is the repression of BAD at 300 μM and 500 μM. Proapoptotic BAD promotes apoptosis by binding to and inhibiting antiapoptotic members of the Bcl‐2 family [63]. The balance between BAX and BCL2 is essential in the regulation of cell survival. It has been reported that U induces a decrease in the mitochondrial concentration of the Bcl‐2 protein in HK‐2 cells treated with 500 μM of DU for 24 h, as well as the overexpression of the Bax level [19]. This regulation in favor of apoptosis with elevation of BAX could be observed at the gene level on hRPTEC TERT1 but could not be confirmed in protein expression (Figure 2). In terms of regulation of survival and apoptosis, an increase in p38‐MAPK phosphorylation and activation could be correlated to apoptosis, notably after uranium exposure on macrophages [22] and on rat bone tissues [64] and for the first time it is observed on the renal epithelial cell hRPTEC TERT1 (Figure 2B). Also noteworthy is the detection by protein array only of increased DDK4 inhibitor of the Wnt/β‐catenin signaling pathway [65], a critical regulator of kidney development that is upregulated after injury and promotes the progression of tubulointerstitial fibrosis [66].

The inflammatory response is highly upregulated on hRPTEC exposed to 500 μM of U(VI): a proinflammatory response, with an elevated cytokine protein level of TNFα, IL‐6, and IL‐18 (Figure 5). TNFα upregulation is also observed in rat renal homogenates from acutely U‐exposed rats [57], in lungs of mice exposed to U [67], and in macrophage cells exposed to U [22]. MIP‐2 overexpression could lead to increased leukocyte migration and inflammation [68] and also appears to be increased for hRPTEC TERT1, as observed in lungs of mice exposed to U [67]. Other markers of the inflammatory response, such as PECAM1, osteopontin (Opn), and Gal‐3 induction, were also induced in mouse kidneys exposed to U in a transcriptomic study [69]. The repression of gene expression of CBS is observed on hRPTEC TERT1 exposed to 500 μM U(VI). This occurrence has been previously shown in rats injected with U, leading to decreasing CBS and cystathionine lyase (CSE) levels, two enzymes that catalyze the endogenous formation of hydrogen sulfide (H_2_S) [57]. H_2_S is an endogenous gaseous signaling molecule that regulates antioxidative, anti‐inflammatory, and cytoprotective responses [70], with downexpression of CBS reducing endogenous H2S formation and contributing to the proinflammatory response. Observing this phenomenon in the hRPTEC model, identified as the primary site for uranium accumulation, provides precise information about the cells involved in this response. At concentrations that cause deleterious effects, a strong proinflammatory response seems to take hold.

With regard to tubular injury, increased levels of KIM‐1, a sensitive biomarker of PCT injury, have previously been observed in murine models treated with acute or repeated exposure of uranium [72]. Few markers of kidney damage are induced in vitro in this study as well as in other renal cell models, despite the cellular specialization of hRPTEC TERT1. Cystatin C and clusterin are relevant functional markers for assessing renal physiology in vivo, but their application to a static cell model is complex, as this model contains only one type of tubular epithelial cell and lacks glomerular filtration. Indeed, clusterin is generally measured in urine as a biomarker of tubular damage, a measurement that is not applicable to our model. Similarly, cystatin C is used clinically as an alternative to creatinine for estimating the glomerular filtration rate. Consequently, the interpretation of results obtained from a tubular epithelial cell model must be done with caution. It might be interesting to study the cellular response in other types of renal cells or in cellular models composed of cells of different types such as organoid models.

Several cell‐based and animal models for uranium nephrotoxicity have been reported in the literature [14, 20, 21, 28, 72]. While animal models, predominantly rodents, have historically been the standard, they present significant limitations. First, interspecies variability in the expression and affinity of renal transporters [73] often hinders the extrapolation of uranium uptake and clearance data to humans. Second, these models involve systemic confounding factors, such as hemodynamics and inflammatory responses, which complicate the isolation of direct cellular toxicity. Finally, ethical constraints (3 Rs) and high operational costs limit their application for large‐scale dose–response screenings.

In this context, our hRPTEC‐based approach offers a highly reproducible, human‐derived platform specifically targeting the proximal tubule—the primary site of uranium accumulation. Key advantages include enhanced physiological relevance by avoiding interspecies transport bias, the ability to decipher specific mechanisms through direct quantification of membrane damage without systemic interference, and superior scalability. Consequently, this model is most appropriately applied as a first‐tier, high‐resolution mechanistic tool to identify early biomarkers and molecular pathways of acute injury prior to validation in complex, systemic environments.

Critically, the present study offers preliminary mechanistic screening that establishes a foundation for future studies. While we have validated several key pathways using targeted approaches such as ELISA and Western blot, we acknowledge that further dedicated studies will be required to fully explore the remaining candidates.

Finaly, the main toxicity of uranium stems from its characteristics as a heavy metal rather than its radioactive properties [74, 75]. However, uranium can induce DNA damage, which is generally attributed to ionizing radiation and thus to the radiological toxicity component. Using different isotopic forms of uranyl nitrate and therefore different specific activities, uranium could induce effects such as mutagenesis [76] and neoplastic transformation [75] of cells, generally associated with radiation. Future studies could investigate the respective roles of radiation and chemical toxicity using exposure to enriched uranium or to alpha particles compared to depleted uranium contamination.

## 5. Conclusion

This exploratory study contributes to the understanding and refining of the key events involved in uranium‐induced nephrotoxicity using a human RPTEC model and to the ongoing development of related AOPs. Using a well‐differentiated human RPTEC model, this work identified specific concentrations of U(VI) associated with deleterious effects, such as apoptosis and cytotoxicity/necrosis, as well as the cellular responses related to oxidative stress, inflammation, and kidney injury (Graphical Abstract). Following the preliminary AOP, uranium is known to alter mitochondrial function, and a resulting rise in ROS and an antioxidant response were observed. U(VI) induced a marked inflammatory response, with dose‐dependent increased levels of TNFα, IL‐6, and IL‐18, sustaining an increase in caspase 3/7 and LDH. These findings underline the importance of considering dose‐dependent effects when assessing the risks associated with exposure to uranium and taking this into account to build the AOP of kidney failure. This research also highlights the importance of using models that are closer to humans, and it would be interesting to complete the current AOP by considering renal impairment with a human 3D in vitro renal model that is even closer to the phenotype and organization of a human kidney, such as kidney organoids [77–78].

## Author Contributions

M.F. and Y.G. proposed the concept of the manuscript and designed the experimental study. M.F., V.P., S.G., A.M., C.S., and Y.G. performed the experiments and collected data. M.F., V.P., S.G., A.M., C.S., T.H., and Y.G. contributed to data analysis and interpretation. M.F., C.S., and Y.G. wrote the manuscript with intellectual input from T.H. and C.B‐C. Supervision and project administration were done by C.S., T.H., and Y.G.

## Funding

This work was supported by ASNR (Autorité de Sûreté Nucléaire et de Radioprotection).

## Disclosure

All authors read and provided input in finalizing the manuscript. The manuscript is part of the thesis of Marie Frerejacques (DOI: 10.70675/56f1d599z39a7z4b8cza415zf0499fbd741d).

## Conflicts of Interest

The authors declare no conflicts of interest.

## Supporting Information

Additional supporting information can be found online in the Supporting Information section.

## Supporting information


**Supporting Information** Supporting Figure S1 “Effect of uranium on the time‐dependent kinetics of LDH activity (necrosis) and 24 h effect on Caspase 3/7 activity” and Figure S2 “Effect of uranium on cytokines secretion” can be uploaded separately. A Graphical Abstract presents a schematic representation of the adverse outcome pathway of kidney toxicity induced by acute exposure to uranium.

## Data Availability

The datasets used and/or analyzed during the current study are available from the corresponding author on request.
