# Supplementary material for: In Vitro hRPTEC TERT1 Model for Uranium‐Induced Nephrotoxicity Pathway Study
Source: J Toxicol. 2026 Jun 17;2026:6692188. doi: 10.1155/jt/6692188 (PMC13276292; doi:10.1155/jt/6692188)
Supplement: Supplementary file 1 — Supporting Information Supporting Figure S1 “Effect of uranium on the time‐dependent kinetics of LDH activity (necrosis) and 24 h effect on Caspase 3/7 activity” and Figure S2 “Effect of uranium on cytokines secretion” can be uploaded separately. A Graphical Abstract presents a schematic representation of the adverse outcome pathway of kidney toxicity induced by acute exposure to uranium. [file JT-2026-6692188-s001.zip › Graphical Abstract Image.pptx]

## Slide 1
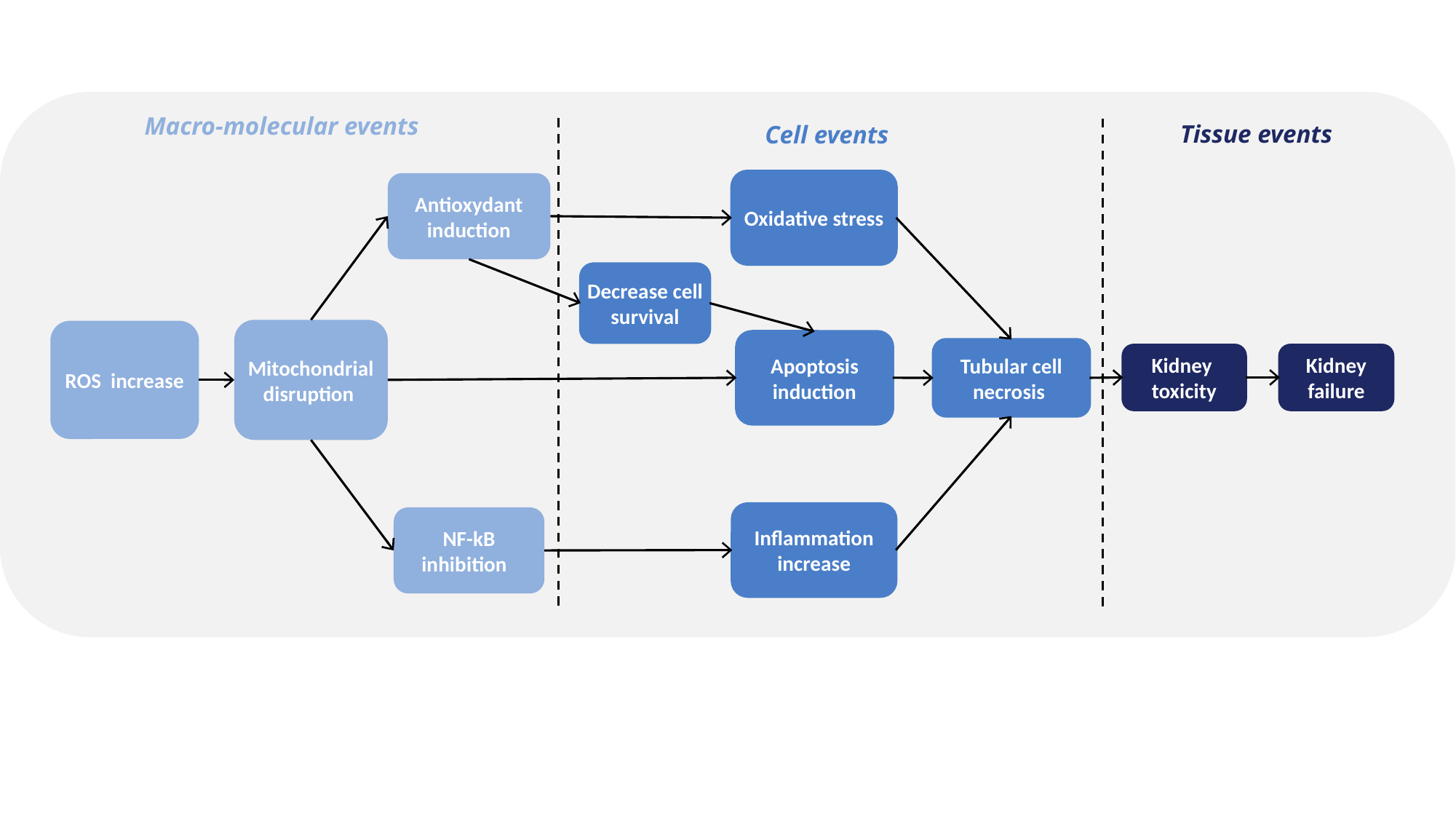

Macro-molecular events
Tissue events
Cell events
Oxidative stress
Antioxydant induction
Decrease cell survival
Mitochondrial disruption
ROS increase
Apoptosis induction
Tubular cell necrosis
Kidney
toxicity
Kidney failure
Inflammation increase
NF-kB inhibition
