# Supplementary material for: In Vitro hRPTEC TERT1 Model for Uranium‐Induced Nephrotoxicity Pathway Study
Source: J Toxicol. 2026 Jun 17;2026:6692188. doi: 10.1155/jt/6692188 (PMC13276292; doi:10.1155/jt/6692188)
Supplement: Supplementary file 1 — Supporting Information Supporting Figure S1 “Effect of uranium on the time‐dependent kinetics of LDH activity (necrosis) and 24 h effect on Caspase 3/7 activity” and Figure S2 “Effect of uranium on cytokines secretion” can be uploaded separately. A Graphical Abstract presents a schematic representation of the adverse outcome pathway of kidney toxicity induced by acute exposure to uranium. [file JT-2026-6692188-s001.zip › Supplementary Material Frerejacques et al rev2.docx]

**Supplementary Material**

LDH and caspase 3/7 assays

##

##

##

*****

*******

***Fig. S1: Effect of uranium on the time-dependant kinetics of LDH activity (necrosis) and 24-hours effect on Caspase 3/7 activity for hRPTEC TERT1 cells***

*Cells were treated with different concentrations of uranium. (A) Cell necrosis was measured by LDH luminescence assay at 2-h, 6-h, 24-h and 48h at increasing concentrations of U(VI). Data were expressed as mean ± SD* where *n=2 per condition. (B) Cell apoptosis was measured by caspase 3/7 luminescence assay. Data were expressed as mean ± SD* where *n=3 per condition. Asterisk represents a significant difference between U(VI) treated and untreated cells (Two-way ANOVA *p<0.05; ** p<0.01; *** p<0.001); Sharp sign represents a significant difference between U(VI) treated concentrations (Two-Way ANOVA #p<0.05; ##p<0.01; ###p<0.001).*

Cytokines assay


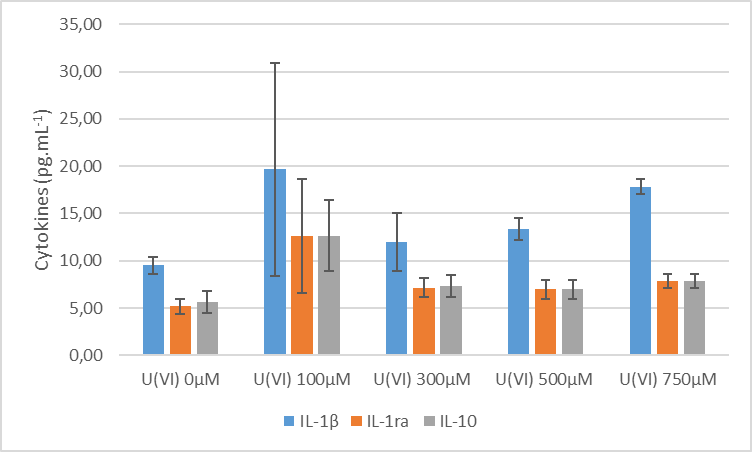


***Fig. S2 Effect of uranium on cytokines secretion by hRPTEC TERT1 cells.*** *Cells were treated with different concentrations of uranium for 48h. IL-1β, IL-1ra and IL-10 protein assay by Milliplex. Data were expressed as mean ± SD (n=3 per condition).*
